# Non-communicable diseases in the world over the past century: a secondary data analysis

**DOI:** 10.3389/fpubh.2024.1436236

**Published:** 2024-10-03

**Authors:** Moslem Taheri Soodejani

**Affiliations:** Center for Healthcare Data Modeling, Department of Biostatistics and Epidemiology, School of Public Health, Shahid Sadoughi University of Medical Sciences, Yazd, Iran

**Keywords:** neurologic disorder, cancer, cardiovascular disease, diabetes mellitus, substance-related disorder, musculoskeletal diseases, global health

## Abstract

**Introduction:**

We analyzed the changes in the top 10 non-communicable diseases (NCDs) over the past century across the World Health Organization (WHO) regions.

**Materials and methods:**

The data were extracted from the Global Burden of Disease (GBD) studies. After we accessed this source, all NCDs were sorted according to their prevalence in 2019, and the 10 most common NCDs were selected. Then, the incidence, prevalence, and mortality rates of these 10 NCDs were compared to the rates in 2000.

**Results:**

Diabetes and kidney disease had the highest increase in incidence (49.4%) and prevalence (28%) in the Eastern Mediterranean region. Substance use disorders had a huge increase (138%) in the mortality rates among women in the Americas region. On the other hand, women in Southeast Asia experienced the greatest decrease in incidence (−19.8%), prevalence (−15.8%), and mortality rates (−66%).

**Conclusion:**

In recent years, nearly all NCDs have shown an increase, yet mortality rates have declined across all regions. Lifestyle can be a major cause of this increase, but advancements in health and medical services, such as screening and treatment, have played a crucial role in improving survival rates.

## Introduction

Non-communicable diseases (NCDs) are one of the biggest global health challenges. Before the COVID-19 pandemic, most studies focused on NCDs; incidence, prevalence, and mortality rates were the topics researchers were interested in when discussing these diseases.

Globally, NCDs are increasing each year, accounting for approximately 60% of all causes of death annually. Notably, approximately 25% of these deaths occur before the age of 60 years. Some studies estimate that approximately 52 million deaths occur around the world each year, with significant variations observed from region to region (1–3).

Lifestyle-related, environmental, and genetic factors are the major risk factors associated with NCDs. Additionally, changes in the population pyramid and globalization contribute to an increased prevalence of these risk factors across all regions (2, 4).

Cardiovascular diseases (CVDs), cancers, respiratory diseases, and diabetes are the most important NCDs, accounting for more than 60% of all deaths (2). Approximately a 30% increase in mortality rates was reported for CVDs as NCDs from 1990 to 2016, and the prevalence of this disease increased by approximately 15% in this same period (5). Chronic respiratory diseases, another group of NCDs, have also seen significant increases, with deaths rising by approximately 18% and prevalence by approximately 40% from 1990 to 2017 (6). Neoplasms are a major type of NCDs; it has been estimated that a 100% increase in new cases has occurred from 2008 to 2022 (12.7 to 22.2 million) (7).

During the COVID-19 pandemic, the WHO focused on COVID-19 to control and manage this challenging situation. However, comorbidities such as hypertension, CADs, chronic kidney disease, malignancies, chronic respiratory disease, and diabetes were the leading causes of COVID-19 deaths and significantly contributed to the increase in mortality rates worldwide (8, 9).

The Sustainable Development Goals (SDGs) have been used by the WHO to transform the world. One of these goals is to promote good health and wellbeing, with a reduction in mortality due to major NCDs as its main target. To achieve this target, we need to have an understanding of major NCDs and examine the changes in incidence, prevalence, and mortality rates in recent years. In this study, we analyzed the changes in the top 10 NCDs over the past century across the WHO regions.

## Materials and methods

### Data source and processing

This study used secondary data from the Institute for Health Metrics and Evaluation. The Global Burden of Diseases (GBD) was a metric that collected and analyzed 87 risk factors and 369 diseases from 204 countries. NCDs were part of this dataset for which incidence, prevalence, and mortality rates were calculated. After access to this source, all the NCDs were sorted according to their prevalence in 2019, and the 10 most common NCDs were selected. These diseases were neurological disorders (Alzheimer’s, Parkinson’s, and multiple sclerosis), musculoskeletal disorders (rheumatoid arthritis), psychological disorders (eating disorder, anorexia nervosa, and bulimia), diabetes and kidney diseases, neoplasms (all cancers), CADs (rheumatic heart disease, ischemic heart disease, stroke, and cardiomyopathy), chronic respiratory diseases (pneumoconiosis, asthma, silicosis, and asbestosis), substance use disorders (alcohol and drug use), skin and subcutaneous diseases (bacterial skin disease and cellulitis), and digestive diseases [chronic liver disease due to viral hepatic or alcohol, peptic ulcer, appendicitis, pancreatic, non-alcoholic fatty liver disease (NAFLD), and non-alcoholic steatohepatitis (NASH)]. The next step was the eradication of these diseases in 2000.

### Data analysis

#### Description of NCDs in 2019

Incidence, prevalence, and mortality rates were the indices used to describe the 10 most common NCDs around the world in 2019. These indices were reported as age-standardized rates per 100,000 populations (95% UI) by sex.

#### Changes in the 10 most common NCDs from 2000 to 2019

To show the changes in the 10 most common NCDs from 2000 to 2019 by sex and WHO regions, Formula 1 was generated and applied to incidence, prevalence, and mortality rates.


(1) $$\%\mathrm{change}=\frac{T1-T0}{T0}*100$$

where T0 is the incidence, prevalence, or mortality rates in 2000, and T1 is the incidence, prevalence, or mortality rates in 2019.

#### Changes in NCDs across the WHO regions

The changes in NCDs (overall) were calculated according to Formula 1. ArcMap GIS 10.1 was used to illustrate the geographical distribution of changes in incidence, prevalence, and mortality rates of NCDs across different WHO regions, based on quartiles (Q1–Q4).

## Results

### Description of NCDs in 2019

#### Results among men

The results showed that skin and subcutaneous diseases (63624.2, 95% UI: 61120.3–63624.2), neurological disorders (9737.5, 95% UI: 8714.8–10772.6), and digestive diseases (5174.1, 95% UI: 4728.2–5,654) had the highest incidence among NCDs, but the most prevalence was reported for neurological disorders (30489.4, 95% UI: 28013.6–32984.1), digestive diseases (29095.9, 95% UI: 27493.5–30652.7), and skin and subcutaneous diseases (25356.9, 95% UI: 24612.5–2649.4). CVDs (280.8, 95% UI: 259.2–299.8), neoplasms (157.1, 95% UI: 144.8–168.3), and chronic respiratory diseases (66.7, 95% UI: 60.5–73.1) had the highest mortality rates.

#### Results among women

The results among women were like those among men. Skin and subcutaneous diseases (61816.9, 95% UI: 59497.7–64330.2), neurological disorders (10784.6, 95% UI: 9731.9–11861.2), and digestive diseases (5733.7, 95% UI: 5237.4–6253.3) had the highest incidence among NCDs. The prevalence of NCDs among women showed that neurological disorders (36401.1, 95% UI: 33706.3–39072.6), skin and subcutaneous diseases (26959.3, 95% UI: 26266.2–27732.4), and digestive diseases (26735.4, 95% UI: 25282.1–28176.7) had the highest prevalence among NCDs.

Like men, CVDs (204, 95% UI: 180.9–221.5), neoplasms (100.5, 95% UI: 92–107.9), and chronic respiratory diseases (39.7, 95% UI: 33.2–44.7) had the highest mortality rates.

Table 1 presents more details.

**Table 1:** Age-standardized rates (ASRs) of incidence, prevalence, and mortality rates of NCDs in the world in 2019.

NCDs	Incidence		Prevalence		Mortality	
	Men	Women	Men	Women	Men	Women
Neurological disorders	9737.5 (8714.8–10772.6)	10784.6 (9731.9–11861.2)	30489.4 (28013.6–32984.1)	36401.1 (33706.3–39072.6)	30.8 (15.3–64.6)	30.3 (12.4–66.9)
Digestive diseases	5174.1 (4728.2–5,654)	5733.7 (5237.4–6253.3)	29095.9 (27493.5–30652.7)	26735.4 (25282.1–28176.7)	40.8 (38–43.8)	24 (21.9–25.9)
Musculoskeletal disorders	(3181–3848.5)	4,368 (3972.8–4797.6)	16121.2 (15216.6–17073.7)	20,601 (19534.5–21705.7)	1.1 (0.9–1.2)	1.9 (1.4–2.3)
Mental disorders	3862.8 (3483.6–4251.4)	5445.3 (4869.8–6051.3)	11727.3 (10835.7–12693.9)	12,760 (11831.7–13763.1)	0 (−)	0 (−)
Diabetes and kidney diseases	514.7 (489.1–543.3)	505.9 (480.1–533.5)	12034.8 (11478.1–12591.2)	12200.9 (11621.5–12775.9)	42.7 (39.6–45.6)	34.1 (30.9–36.8)
Neoplasms	3457.3 (2948–4109.4)	4116.5 (3461.9–4918.4)	5287.5 (4570.5–6108.1)	6,696 (5754.9–7759.9)	157.1 (144.8–168.3)	100.5 (92–107.9)
Cardiovascular diseases	729.8 (687.7–774.5)	642.7 (607.2–680.1)	6471.7 (6136.7–6814.4)	6402.6 (6078.9–6,740)	280.8 (259.2–299.8)	204 (180.9–221.5)
Chronic respiratory diseases	1034.1 (910–1187.4)	973.4 (860.6–1108.6)	5907.7 (5382–6581.2)	5695.9 (5209.3–6282.1)	66.7 (60.5–73.1)	39.7 (33.2–44.7)
Substance use disorders	1239.7 (1065–1432.1)	486.1 (421.4–561.1)	2831.4 (2544.6–3152.9)	1167.6 (1050.3–1315.1)	5.7 (5.2–6.1)	1.5 (1.4–1.6)
Skin and subcutaneous diseases	63624.2 (61120.3–63624.2)	61816.9 (59497.7–64330.2)	25356.9 (24612.5–2649.4)	26959.3 (26266.2–27732.4)	1.31 (0.82–1.82)	1.29 (0.96–1.62)

### Changes in the top 10 NCDs from 2000 to 2019

#### Changes in incidence

**Figure 1:**
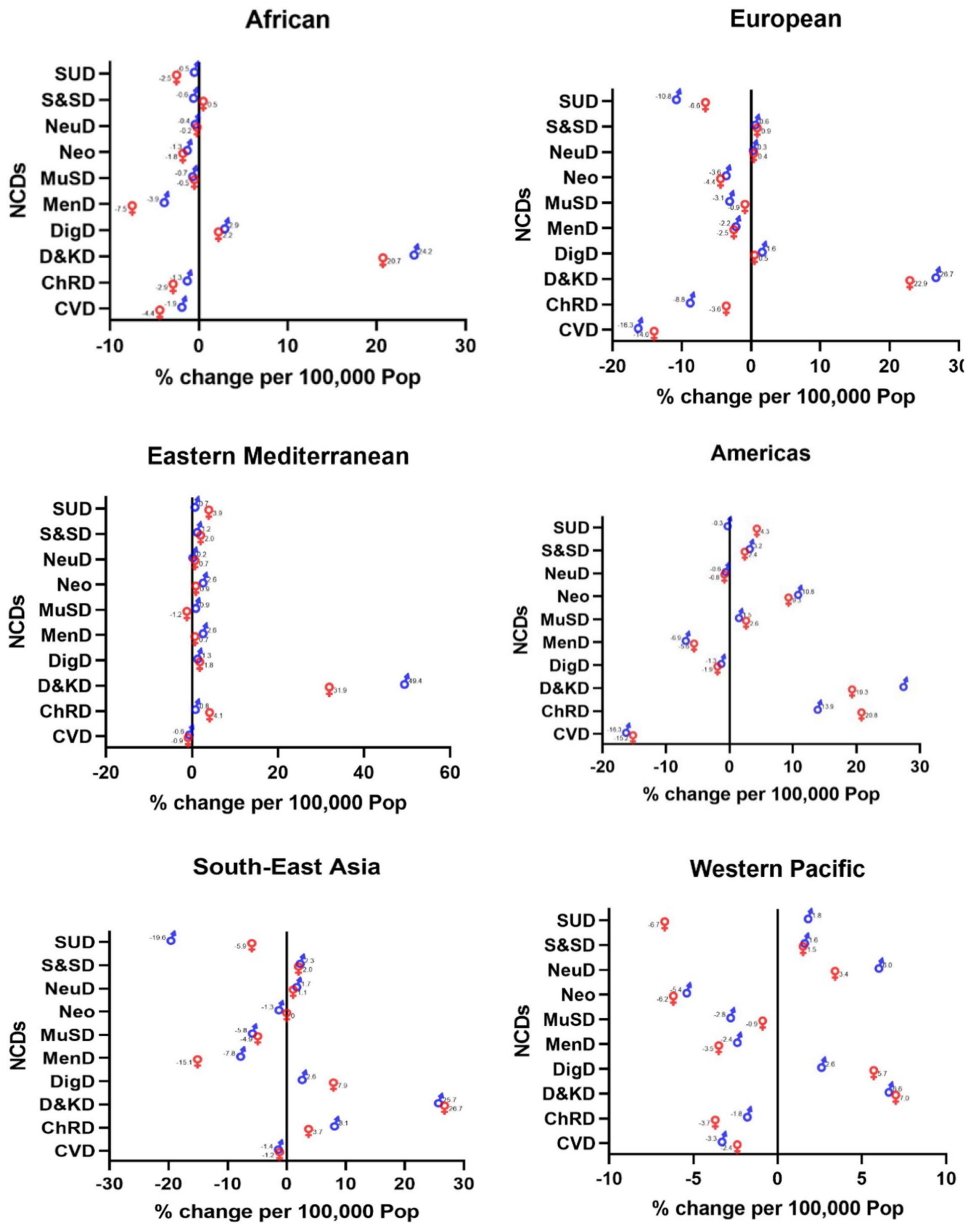
Changes in the incidence of NCDs across the WHO regions (2019 compared to 2000).

Diabetes and kidney diseases are two NCDs that have had the highest increase in recent years. The rise occurred across all WHO regions, and it was the highest among Eastern Mediterranean men (49.4%), American women (28%), European men (26.7%), Southeast Asian women (26.7%), African men (24.2%), and Western Pacific women (7%) (Figure 1).

Substance use disorders among Southeast Asian men (−19.6%), CADs among European men (−16.3%) and American women (−16.3%), mental disorders among African women (−7.5%), substance use disorders among Western Pacific women (−6.7%), and musculoskeletal disorders among Eastern Mediterranean women (−1.2%) had the highest decrease (Figure 1).

#### Changes in prevalence

**Figure 2:**
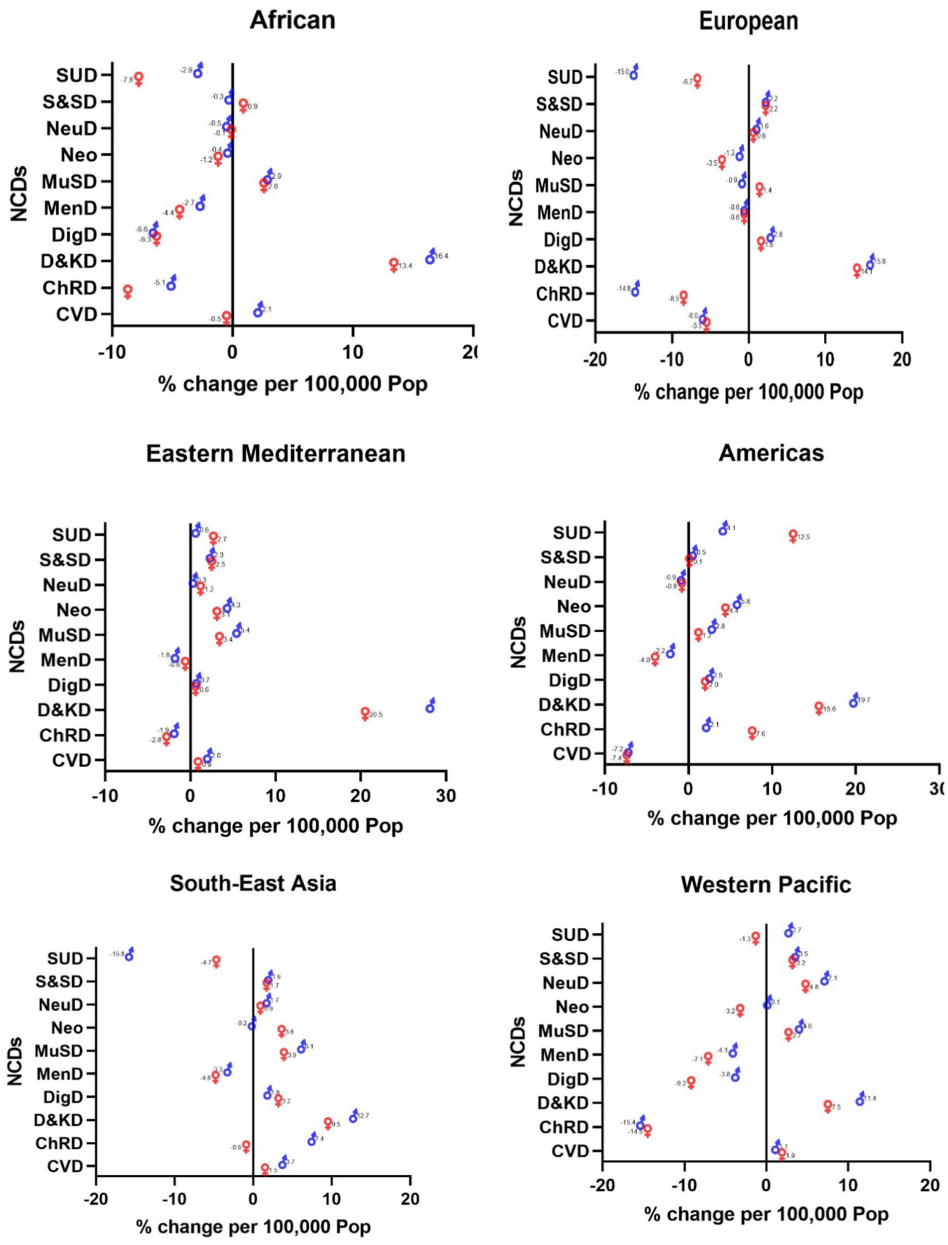
Changes in the prevalence of NCDs across the WHO regions (2019 compared to 2000).

Likewise, the incidence of diabetes and kidney diseases was on the rise among NCDs. Eastern Mediterranean men (28%), American men (19.7%), African men (16.4%), European men (15.8%), Southeast Asian men (12.7%), and Western Pacific men (11.4%) showed the most increase (Figure 2).

Substance use disorders among Southeast Asian women (−15.8%), chronic respiratory diseases among Western Pacific men (−15.4%), substance use disorders among European men (−15%), substance use disorders among African men (−7.8%), CVDs among American women (−7.4%), and chronic respiratory diseases among Eastern Mediterranean men (−2.8%) had the highest decrease (Figure 2).

#### Changes in death

**Figure 3:**
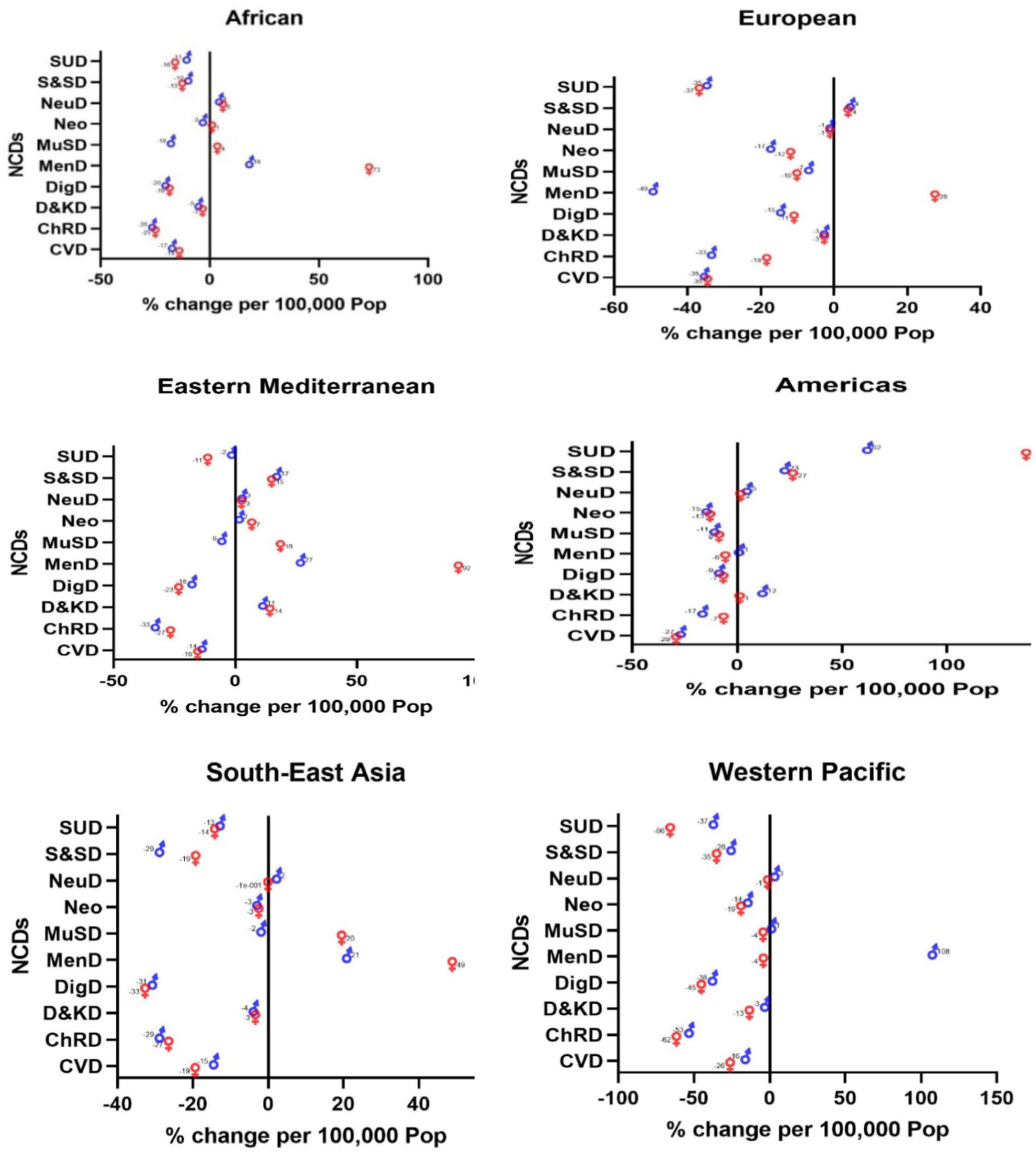
Changes in mortality rates due to NCDs across the WHO regions (2019 compared to 2000).

Substance use disorders among American women saw a huge increase of 138%. Mental disorders were the second most significant NCD to increase in recent years; with the following increases reported: Western Pacific women (108%), Eastern Mediterranean women (92%), African women (73%), Southeast Asian women (49%), and European women (28%), (Figure 3).

Contrary to other regions, substance use disorders had the greatest reduction among Southeast Asian women (−66%), and mental disorders had the highest decrease among women in the European region (−49%). Chronic respiratory diseases were another NCD that saw a decrease among Eastern Mediterranean men (−33%). Digestive disease among Southeast Asian women (−33), CVDs among American women (−29%), and chronic respiratory diseases among African men (−26%) decreased as well (Figure 3).

### Changes in NCDs across the WHO regions

#### Changes in incidence

**Figure 4:**
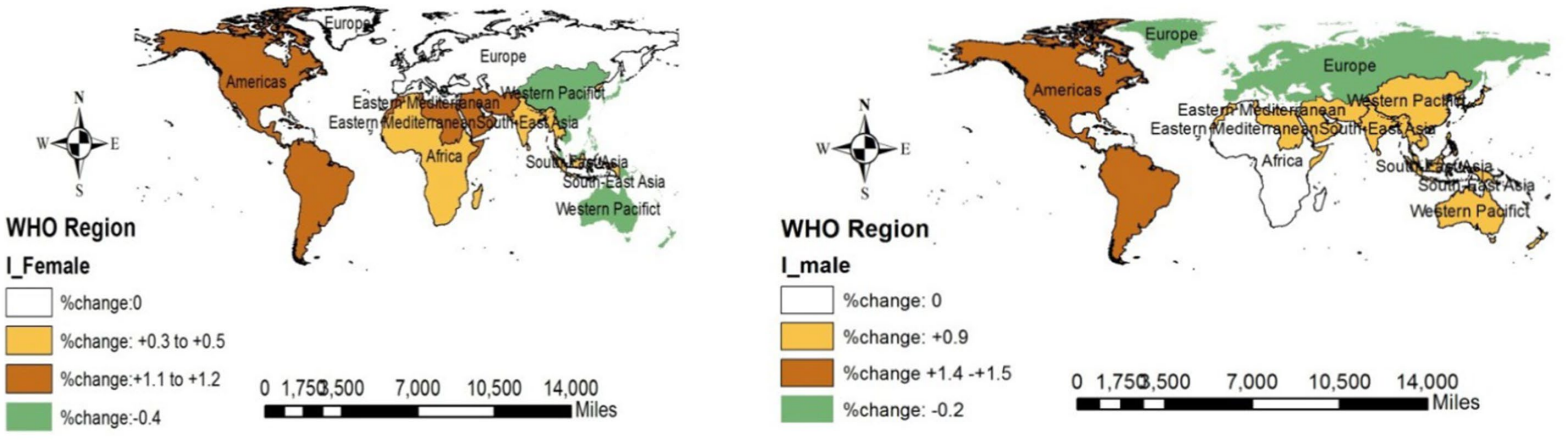
Changes in the incidence of NCDs (overall) across the WHO regions (2019 compared to 2000).

Western Pacific women reported a decreasing change in all NCDs (−0.4%), but this factor increased in American women (1.1–1.2%). Like women, men in the Western Pacific region showed a decreasing change (−0.2%), but this value in the Americas region was on the rise (1.4–1.5%). Figure 4 shows the overall changes in NCDs across the WHO regions.

#### Changes in prevalence

**Figure 5:**
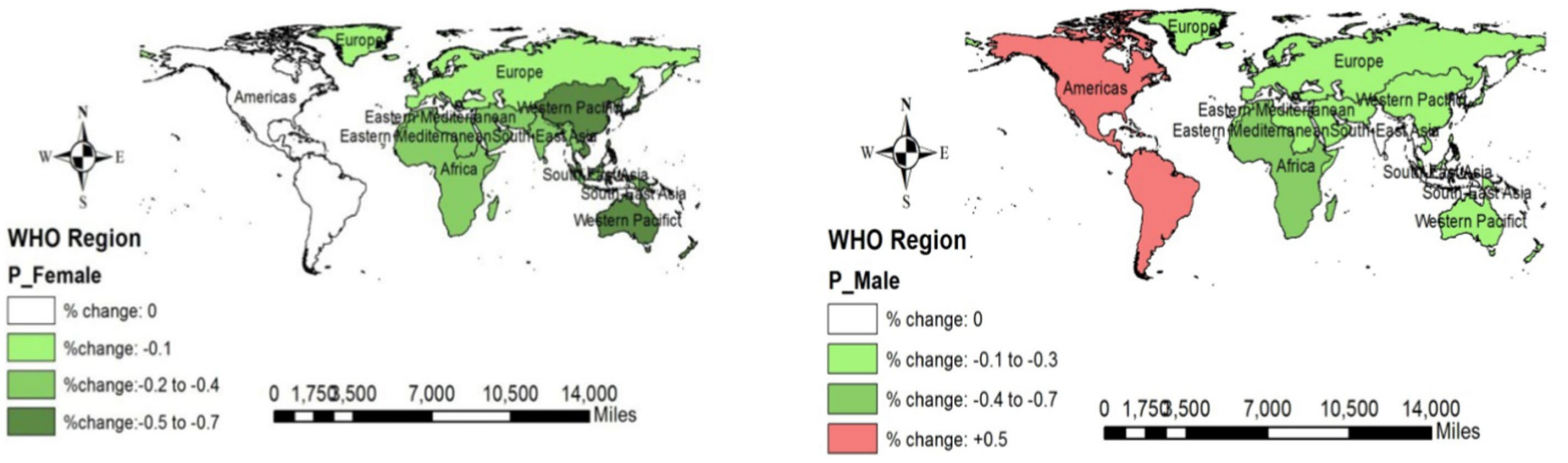
Changes in the prevalence of NCDs (overall) across the WHO regions (2019 compared to 2000).

The prevalence of NCDs in all WHO regions was decreasing among men, but across the Americas region, it was increasing (0.5%). Figure 5 depicts the overall NCD changes across the WHO regions.

#### Changes in mortality rates

**Figure 6:**
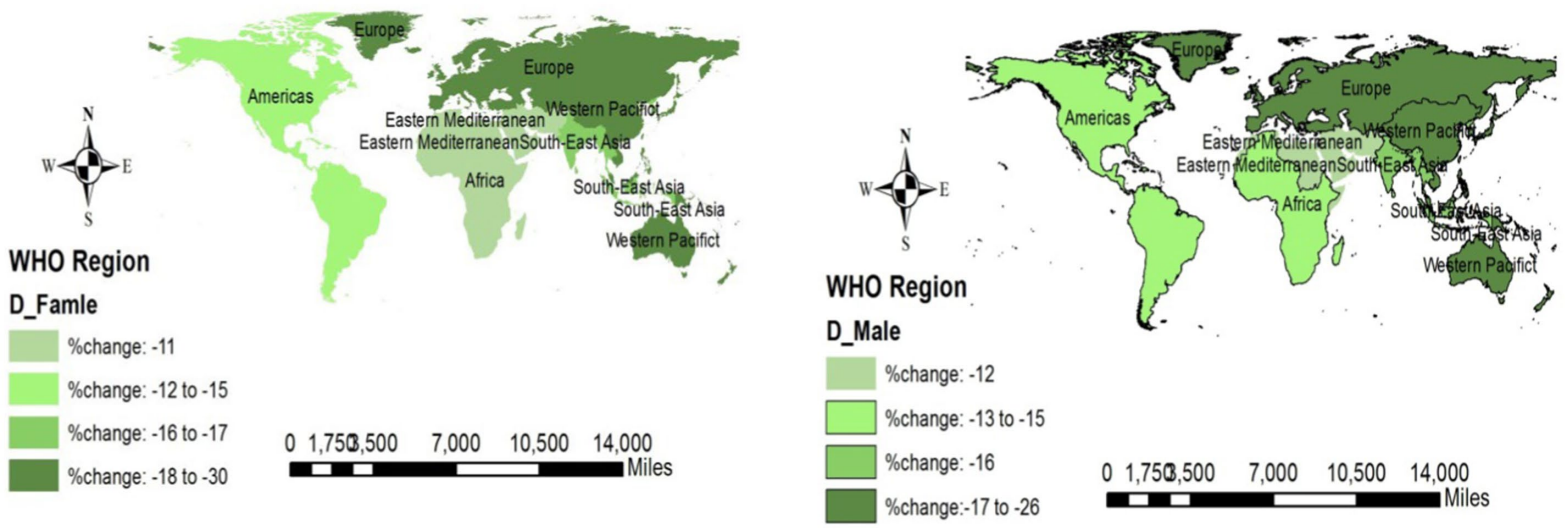
Changes in death due to NCDs (overall) across the WHO regions (2019 compared to 2000).

Fortunately, the mortality rates of NCDs across all regions and both sexes were decreasing. European and Western Pacific regions reported the highest decrease (−18% to −30% among men and −17 to 26% among women) across the WHO regions. More details are displayed in Figure 6.

## Discussion

An overview of the results shows that the incidence of substance use disorders and diabetes and kidney diseases has increased across all WHO regions; the prevalence of mental disorders, diabetes and kidney diseases, and substance use disorders has increased across all WHO regions as well. On the other hand, the mortality rates due to digestive diseases, chronic respiratory diseases, CVDs, and neurological disorders have decreased across all WHO regions. We will clarify the probable causes of the changes in incidence, prevalence, and mortality rates associated with NCDs.

### Neurological disorders

The incidence and prevalence of this disease have increased in Europe, Southeast Asia, and the Western Pacific region; this increase was observed in the mortality rates in Africa, the Eastern Mediterranean, and the American region. Aging and the quality of rehabilitation and treatment services could be major causes of this difference among regions. Furthermore, measurement bias because of self-reporting and variations in the definitions of certain neurological disorders, such as dementia, multiple sclerosis, and Parkinson’s disease, could have led to differences in the rates of these diseases across different regions (10, 11).

### Digestive diseases

Except for the Americas, the rest of the regions showed an increased incidence of digestive diseases. Contrary to this result, the prevalence of digestive diseases has decreased in the Americas and Eastern Mediterranean region. The mortality rates from digestive diseases in all the regions have declined. A wide range of digestive diseases, including chronic liver disease due to viral hepatitis or alcohol, peptic ulcer, appendicitis, NAFLD, and NASH, are influenced by several risk factors such as lifestyle choices and psychological attributes. One of the reasons for the high prevalence of digestive diseases in the Americas could be lifetime chronic digestive diseases in this region (12, 13).

### Musculoskeletal disorders

The incidence and prevalence of this disorder have been growing in recent years. In contrast, the mortality rates have decreased. Aging and a sedentary lifestyle are two major risk factors for these disorders. Additionally, changes in the workforce and the increasing number of workers over 65 years old have played a role in the rise of these conditions during the specified period (14–16). Long-term stress, lack of social support, depression, and sleep problems are the other major risk factors (17).

### Mental disorders

The incidence and prevalence of mental disorders have dropped in almost all regions, but mortality rates have risen in almost all regions. Since these results focused on eating disorders while major psychological disorders such as depression, anxiety, autism, and schizophrenia were missed, the major causes of this outcome could be eating disorders, obesity, weight stigma, dieting, and starvation (18, 19).

### Diabetes and kidney diseases

The prevalence and incidence of diabetes and kidney diseases have been increasing in recent years worldwide; the Eastern Mediterranean is a region that has witnessed the biggest change. This trend could be expected due to an increase in sedentary lifestyles, food habits, and smoking in almost all countries; on the other hand, since diabetes and kidney diseases are chronic conditions, their prevalence tends to increase every year (20–22).

### Neoplasms

The prevalence, incidence, and mortality rates have dropped in almost all regions. This improvement can be attributed to factors such as screening programs, early detection, high education levels, and better health service promotion. However, it is important to note that the situation varies by country; while some cancers are decreasing, others are increasing in different regions. For example, breast cancer is one of the most prevalent neoplasms in many countries, but screening programs have helped in the early detection and contributed to a reduction in mortality rates of this cancer (7, 23–25). Moreover, many countries within a region have varying levels of income, and these differences can affect the prevalence, incidence, and mortality rates of neoplasms in the region (23).

### Cardiovascular diseases

The incidence of CVDs has decreased, while the prevalence is still high; mortality rates have decreased in all regions as well. Overall, CVDs have a high prevalence around the world because of lifestyle changes. Nevertheless, CVDs are increasing in developing countries and declining in developed countries. It seems that some countries have achieved the SDGs by reducing CVD deaths, which is a main goal of NCDs (5, 26).

### Chronic respiratory diseases

The prevalence and incidence across the Americas, Southeast Asia, and Eastern Mediterranean regions have increased; in contrast, mortality rates have recently decreased. Smoking, household pollution due to solid fuel, occupational and biomass exposures, and urbanization could be causes of these results. In addition, an increase in non-fatal respiratory diseases and health service promotion could have reduced the global mortality rates (27–30).

### Substance use disorders

Except for the Americas, in the rest of the regions, incidence, prevalence, and mortality rates have declined. It is difficult to find reasons for the increasing substance use in America, but the legalization of some substances such as cannabis in many states and their normalization may justify these results (31).

### Skin and subcutaneous diseases

Cellulitis has increased in the specified years in almost all regions. Several studies showed that the high prevalence of injury, smoking, overweight, and diabetes mellitus could increase cellulitis. These risk factors have risen across all communities, contributing to the rising prevalence of these diseases (27, 28, 32–34).

## Conclusion

Almost all NCDs have increased in recent years; despite this, the mortality rates have declined in all. Lifestyle choices can be a major cause of the increase, but health and medical services such as screening and treatment help people survive. On the other hand, according to prevalence, incidence, and disease duration (P = I*D), even if the incidence does not change, the prevalence will remain high for many years. Although survival is preferred, living with a disease for a lifetime may not be the best choice. *Prevention is better than cure* is a slogan that we should strive toward.

### Strengths and limitations

This was a comprehensive study in which the most common NCDs across the WHO regions were assessed. These results have been reported by regions and NCDs and discussed point by point. Additionally, this was an ecological study with the potential for ecological fallacy; thus, we could not attribute the results of a region to every country in that region. In addition, this is just a descriptive study that has not used analytical statistics.

## Summary

What is already known on this topic?

It is widely recognized that NCDs are a significant global health concern, responsible for many deaths each year.

What is added by this report?

This research shows the changes in incidence, prevalence, and mortality rates for every NCD across the WHO regions.

What are the implications for public health practice?

Updated information about NCDs could help health policymakers design an appropriate intervention to prevent NCDs by region.

## Data Availability

Publicly available datasets were analyzed in this study. This data can be found at: https://vizhub.healthdata.org/gbd-results/.

## References

[1] Jan S Laba T-L Essue BM Gheorghe A Muhunthan J Engelgau M. Action to address the household economic burden of non-communicable diseases. Lancet. (2018) 391:2047–58. doi: 10.1016/S0140-6736(18)30323-4, PMID: 29627161

[2] Bhandari GP Angdembe MR Dhimal M Neupane S Bhusal C. State of non-communicable diseases in Nepal. BMC Public Health. (2014) 14:1–9. doi: 10.1186/1471-2458-14-23 24405646 PMC3893427

[3] Boutayeb A Boutayeb S Boutayeb W. Multi-morbidity of non-communicable diseases and equity in WHO eastern Mediterranean countries. Int J Equity Health. (2013) 12:1–13. doi: 10.1186/1475-9276-12-60 23961989 PMC3848740

[4] Maiyaki MB Garbati MA. The burden of non-communicable diseases in Nigeria; in the context of globalization. Ann Afr Med. (2014) 13:1–10. doi: 10.4103/1596-3519.126933, PMID: 24521570

[5] Liu S Li Y Zeng X Wang H Yin P Wang L. Burden of cardiovascular diseases in China, 1990-2016: findings from the 2016 global burden of disease study. JAMA Cardiol. (2019) 4:342–52. doi: 10.1001/jamacardio.2019.0295, PMID: 30865215 PMC6484795

[6] Soriano JB Kendrick PJ Paulson KR Gupta V Abrams EM Adedoyin RA. Prevalence and attributable health burden of chronic respiratory diseases, 1990–2017: a systematic analysis for the global burden of disease study 2017. Lancet Respir Med. (2020) 8:585–96. doi: 10.1016/S2213-2600(20)30105-3, PMID: 32526187 PMC7284317

[7] Bray F Jemal A Grey N Ferlay J Forman D. Global cancer transitions according to the human development index (2008–2030): a population-based study. Lancet Oncol. (2012) 13:790–801. doi: 10.1016/S1470-2045(12)70211-5, PMID: 22658655

[8] Djaharuddin I Munawwarah S Nurulita A Ilyas M Tabri NA Lihawa N. Comorbidities and mortality in COVID-19 patients. Gac Sanit. (2021) 35:S530–2. doi: 10.1016/j.gaceta.2021.10.085, PMID: 34929892 PMC8677356

[9] Sanyaolu A Okorie C Marinkovic A Patidar R Younis K Desai P. Comorbidity and its impact on patients with COVID-19. SN Compr Clin Med. (2020) 2:1069–76. doi: 10.1007/s42399-020-00363-4, PMID: 32838147 PMC7314621

[10] Feigin VL Nichols E Alam T Bannick MS Beghi E Blake N. Global, regional, and national burden of neurological disorders, 1990–2016: a systematic analysis for the global burden of disease study 2016. Lancet Neurol. (2019) 18:459–80. doi: 10.1016/S1474-4422(18)30499-X, PMID: 30879893 PMC6459001

[11] Leonardi M Steiner TJ Scher AT Lipton RB. The global burden of migraine: measuring disability in headache disorders with WHO's classification of functioning, disability and health (ICF). J Headache Pain. (2005) 6:429–40. doi: 10.1007/s10194-005-0252-4, PMID: 16388337 PMC3452308

[12] Stanghellini V Chan FK Hasler WL Malagelada JR Suzuki H Tack J. Gastroduodenal disorders. Gastroenterology. (2016) 150:1380–92. doi: 10.1053/j.gastro.2016.02.011, PMID: 27147122

[13] Lewis JR Mohanty SR. Non-alcoholic fatty liver disease: a review and update. Dig Dis Sci. (2010) 55:560–78. doi: 10.1007/s10620-009-1081-0, PMID: 20101463

[14] Vos T Barber RM Bell B Bertozzi-Villa A Biryukov S Bolliger I. Global, regional, and national incidence, prevalence, and years lived with disability for 301 acute and chronic diseases and injuries in 188 countries, 1990–2013: a systematic analysis for the global burden of disease study 2013. Lancet. (2015) 386:743–800. doi: 10.1016/S0140-6736(15)60692-4, PMID: 26063472 PMC4561509

[15] Davis K Dunning K Jewell G Lockey J. Cost and disability trends of work-related musculoskeletal disorders in Ohio. Occup Med. (2014) 64:608–15. doi: 10.1093/occmed/kqu126, PMID: 25298392

[16] Wang K Xing D Dong S Lin J. The global state of research in non-surgical treatment of knee osteoarthritis: a bibliometric and visualized study. BMC Musculoskelet Disord. (2019) 20:1–10. doi: 10.1186/s12891-019-2804-9 31484517 PMC6727547

[17] Kazeminasab S Nejadghaderi SA Amiri P Pourfathi H Araj-Khodaei M Sullman MJ. Neck pain: global epidemiology, trends and risk factors. BMC Musculoskelet Disord. (2022) 23:1–13. doi: 10.1186/s12891-021-04957-4 34980079 PMC8725362

[18] Hilbert A Pike KM Goldschmidt AB Wilfley DE Fairburn CG Dohm F-A. Risk factors across the eating disorders. Psychiatry Res. (2014) 220:500–6. doi: 10.1016/j.psychres.2014.05.054, PMID: 25103674 PMC4785871

[19] Striegel-Moore RH Bulik CM. Risk factors for eating disorders. Am Psychol. (2007) 62:181. doi: 10.1037/0003-066X.62.3.181, PMID: 17469897

[20] Koye DN Magliano DJ Nelson RG Pavkov ME. The global epidemiology of diabetes and kidney disease. Adv Chronic Kidney Dis. (2018) 25:121–32. doi: 10.1053/j.ackd.2017.10.011, PMID: 29580576 PMC11000253

[21] Gheith O Farouk N Nampoory N Halim MA Al-Otaibi T. Diabetic kidney disease: world wide difference of prevalence and risk factors. J Nephropharmacol. (2016) 5:49. PMID: 28197499 PMC5297507

[22] Harding JL Pavkov ME Magliano DJ Shaw JE Gregg EW. Global trends in diabetes complications: a review of current evidence. Diabetologia. (2019) 62:3–16. doi: 10.1007/s00125-018-4711-2, PMID: 30171279

[23] Malvezzi M Carioli G Bertuccio P Boffetta P Levi F La Vecchia C. European cancer mortality predictions for the year 2019 with focus on breast cancer. Ann Oncol. (2019) 30:781–7. doi: 10.1093/annonc/mdz051, PMID: 30887043

[24] Chatenoud L Bertuccio P Bosetti C Malvezzi M Levi F Negri E. Trends in mortality from major cancers in the Americas: 1980–2010. Ann Oncol. (2014) 25:1843–53. doi: 10.1093/annonc/mdu206, PMID: 24907637

[25] Fitzmaurice C Dicker D Pain A Hamavid H Moradi-Lakeh M Mac Intyre MF. The global burden of cancer 2013. JAMA Oncol. (2015) 1:505–27. doi: 10.1001/jamaoncol.2015.0735, PMID: 26181261 PMC4500822

[26] Gupta R Mohan I Narula J. Trends in coronary heart disease epidemiology in India. Ann Glob Health. (2016) 82:307–15. doi: 10.1016/j.aogh.2016.04.002 27372534

[27] Chlebicki MP Oh CC. Recurrent cellulitis: risk factors, etiology, pathogenesis and treatment. Curr Infect Dis Rep. (2014) 16:1–8. doi: 10.1007/s11908-014-0422-0 24980389

[28] Cannon J Dyer J Carapetis J Manning L. Epidemiology and risk factors for recurrent severe lower limb cellulitis: a longitudinal cohort study. Clin Microbiol Infect. (2018) 24:1084–8. doi: 10.1016/j.cmi.2018.01.023, PMID: 29427799

[29] Simonsen SE Van Orman E Hatch B Jones S Gren L Hegmann K. Cellulitis incidence in a defined population. Epidemiol Infect. (2006) 134:293–9. doi: 10.1017/S095026880500484X, PMID: 16490133 PMC2870381

[30] Andersen PK Geskus RB de Witte T Putter H. Competing risks in epidemiology: possibilities and pitfalls. Int J Epidemiol. (2012) 41:861–70. doi: 10.1093/ije/dyr213, PMID: 22253319 PMC3396320

[31] Pathak S Deruiter J Ramesh S Govindarajulu M Almaghrabi M Nadar R. The legality of use and consumption of cannabis (marijuana) in the United States of America. Singapore: Cannabis/Marijuana for Healthcare, Springer; (2022). 113–130.

[32] Vignes S Poizeau F Dupuy A. Cellulitis risk factors for patients with primary or secondary lymphedema. J Vasc Surg Venous Lymphat Disord. (2022) 10:179–85. e1. doi: 10.1016/j.jvsv.2021.04.009, PMID: 33957278

[33] Halilovic J Heintz BH Brown J. Risk factors for clinical failure in patients hospitalized with cellulitis and cutaneous abscess. J Infect. (2012) 65:128–34. doi: 10.1016/j.jinf.2012.03.013, PMID: 22445732

[34] Teerachaisakul M Ekataksin W Durongwatana S Taneepanichskul S. Risk factors for cellulitis in patients with lymphedema: a case-controlled study. Lymphology. (2013) 46:150–6. PMID: 24645538

